# Can Bangladesh produce enough cereals to meet future demand?

**DOI:** 10.1016/j.agsy.2016.11.003

**Published:** 2018-06

**Authors:** J. Timsina, J. Wolf, N. Guilpart, L.G.J. van Bussel, P. Grassini, J. van Wart, A. Hossain, H. Rashid, S. Islam, M.K. van Ittersum

**Affiliations:** a Faculty of Veterinary and Agricultural Sciences, University of Melbourne, Victoria 3010, Australia; b Plant Production Systems, Wageningen University, P.O. Box 430, 6700 AK Wageningen, Netherlands; c Department of Agronomy and Horticulture, University of Nebraska-Lincoln, Lincoln, NE 68583, USA; d Wheat Research Centre, Bangladesh Agriculture Research Institute, Dinajpur, Bangladesh; e Bangladesh Rice Research Institute, Gazipur, Bangladesh; f International Maize and Wheat Improvement Center — Bangladesh, House 10/B, Road 53, Gulshan-2, Dhaka 1213, Bangladesh

**Keywords:** Food security, Yield potential, Yield gap, Self-sufficiency ratio, Cropland area, Land use change scenarios

## Abstract

Bangladesh faces huge challenges in achieving food security due to its high population, diet changes, and limited room for expanding cropland and cropping intensity. The objective of this study is to assess the degree to which Bangladesh can be self-sufficient in terms of domestic maize, rice and wheat production by the years 2030 and 2050 by closing the existing gap (Yg) between yield potential (Yp) and actual farm yield (Ya), accounting for possible changes in cropland area. Yield potential and yield gaps were calculated for the three crops using well-validated crop models and site-specific weather, management and soil data, and upscaled to the whole country. We assessed potential grain production in the years 2030 and 2050 for six land use change scenarios (general decrease in arable land; declining ground water tables in the north; cropping of fallow areas in the south; effect of sea level rise; increased cropping intensity; and larger share of cash crops) and three levels of Yg closure (1: no yield increase; 2: Yg closure at a level equivalent to 50% (50% Yg closure); 3: Yg closure to a level of 85% of Yp (irrigated crops) and 80% of water-limited yield potential or Yw (rainfed crops) (full Yg closure)). In addition, changes in demand with low and high population growth rates, and substitution of rice by maize in future diets were also examined. Total aggregated demand of the three cereals (in milled rice equivalents) in 2030 and 2050, based on the UN median population variant, is projected to be 21 and 24% higher than in 2010. Current Yg represent 50% (irrigated rice), 48–63% (rainfed rice), 49% (irrigated wheat), 40% (rainfed wheat), 46% (irrigated maize), and 44% (rainfed maize) of their Yp or Yw. With 50% Yg closure and for various land use changes, self-sufficiency ratio will be > 1 for rice in 2030 and about one in 2050 but well below one for maize and wheat in both 2030 and 2050. With full Yg closure, self-sufficiency ratios will be well above one for rice and all three cereals jointly but below one for maize and wheat for all scenarios, except for the scenario with drastic decrease in boro rice area to allow for area expansion for cash crops. Full Yg closure of all cereals is needed to compensate for area decreases and demand increases, and then even some maize and large amounts of wheat imports will be required to satisfy demand in future. The results of this analysis have important implications for Bangladesh and other countries with high population growth rate, shrinking arable land due to rapid urbanization, and highly vulnerable to climate change.

## Highlights

• Relative yield gap (Yg) across water regime is around 45% (maize and wheat) and 50-60% (rice) in Bangladesh.
• Relative Yg of three cereals is about 46-50% (under irrigation) and 40-63% (under rainfed).
• With 50% Yg closure, self-sufficiency ratio (SSR) in 2050 will be ~ 1 (rice) but significantly < 1 (maize and wheat).
• With full Yg closure, SSR in 2050 will be greater than 1 (rice) but less than 1 (maize and wheat).
• Full Yg closure in future can compensate area decrease in rice but some maize and large wheat imports will be inevitable.

## 1 Introduction

Bangladesh is a deltaic country located in South Asia, with a relatively small land area (147,570 km^2^) but with the 8th largest world population (ca. 161 million) and the 13th highest world population density. According to the medium variant UN projection (UN, 2015), Bangladesh’ population will further increase to 186 and 202 million by the years 2030 and 2050, respectively. Increasing income level and urbanization may lead to diet changes such as switching from traditional rice to wheat and to livestock, poultry, and fish products, which in turn require large amounts of maize for their production (Alkanda, 2010, Mukherjee et al., 2011). Most land suitable for cropping in the country is already under cultivation. Arable land area is even decreasing over time due to increasing demand for residential and industrial use (Hasan et al., 2013). Bangladesh also suffers from periodic natural calamities such as drought, flooding, and cyclones. Due to its location in a delta, climate change and associated sea level rise is expected to increase risk for flooding and salinization of agricultural lands, especially near the southern coast (Hossain and Silva, 2013, MOA-FAO, 2012).

In irrigated cropping systems, yield potential (Yp) is defined as the yield of an adapted crop cultivar when grown without water and nutrient limitations and kept free of biotic stresses (Evans, 1993). In rainfed systems, water-limited yield potential (Yw) is determined by the soil water availability as governed by the water supply amount and distribution and soil and terrain properties, and no nutrient limitation and free of biotic stresses (van Ittersum et al., 2013). Yield gap (Yg) is defined as the difference between Yp (irrigated systems) or Yw (rainfed systems) and actual farm yield (Ya) (Van Ittersum and Rabbinge, 1997, Van Ittersum et al., 2013). Although cereal yields in Bangladesh have increased substantially over time, previous studies have documented large Yg in farmers' fields (Hasan and Islam, 2010, Kashem et al., 2012, Khan et al., 2013, Mondal, 2011, Schulthess et al., 2012, Timsina et al., 2010), though these studies were limited to a few locations and did not look at the potential extra grain production in Bangladesh due to Yg closure.

Current production-consumption ratio, hereafter called self-sufficiency ratio (SSR) is 1.09, 0.21, 0.67 for rice, wheat and maize, respectively (FAOSTAT, 2015), indicating that Bangladesh is self-sufficient in rice, but not in wheat and maize, and consequently highly dependent on trade. Given limited room for cropland expansion, there are basically two options to meet future increase in grain demand without increasing reliance on food imports: (i) increasing cereal yields per ha by closing the existing Yg between potential and actual yield, and (ii) increasing cropping intensity (number of crops planted in the same piece of land during a 12-month period). However, since average cropping intensity in Bangladesh is already high (at least two crops per year); it can be hypothesized that future SSRs will depend on the degree of Yg closure on existing land area (Ahmed et al., 2013, BBS (Bangladesh Bureau of Statistics), 2013). Approaching 80–85% of Yp or Yw, which are considered to be attainable farm yields under good farm management (Cassman et al., 2003, Van Wart et al., 2013), could be an important strategy towards meeting the future food consumption needs of Bangladesh.

Several studies on food security have been conducted for Bangladesh (e.g., Amarasinghe et al., 2014, Ganesh-Kumar et al., 2012, Mainuddin and Kirby, 2015). All these studies, however, considered only one or two crops (typically rice and/or wheat) for a limited number of locations. Likewise, they did not account for potential changes in land use due to urbanization, climate change or other factors. Perhaps more important, these previous estimates of Yg are biased because Yp or Yw were calculated from highest-yielding treatments in research farms (Ali et al., 2008, Hasan and Islam, 2010, Kashem et al., 2012, Khan et al., 2013, Mondal, 2011). Well-validated crop simulation models, coupled with local weather, soil, and management data can provide more robust estimates of average Yp, Yw, and Yg because these models can account for major environment x management x genotype interactions (Van Ittersum et al., 2013, Grassini et al., 2015). With the proper spatial framework, estimates of Yp, Yw, and Yg can be upscaled to larger spatial domains (Van Wart et al., 2013, van Bussel et al., 2015) and serve as foundation for assessing food security scenarios (e.g., van Oort et al., 2015).

Performing a solid food security analysis for Bangladesh is important due to its high population, limited room for cropland expansion, and vulnerability to climate change. Results of this analysis can be used by policymakers to prioritize further research and/or to focus on regions with high potential production. The methodology applied in this study will also be relevant for other regions of the world where population is high, cropland area expansion is not possible, and climate change impact is predicted to be substantial. The objective of this paper is to assess the degree to which Bangladesh can be self-sufficient for maize, rice and wheat by years 2030 and 2050 for different levels of Yg closure, accounting for changes in cropland area, irrigation development, and changes in the relative share of cropland area among the different crop species.

## 2 Materials and methods

### 2.1 Cropping system features

**Fig. 1:**
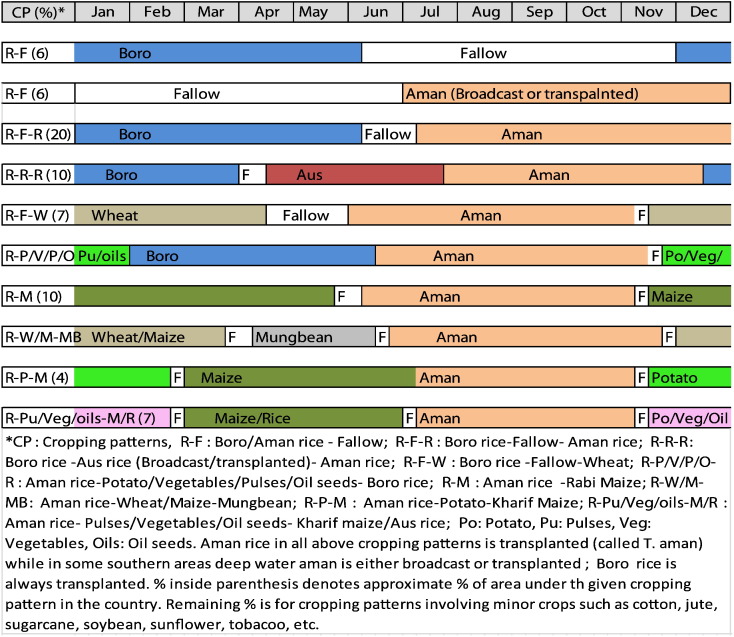
Major cropping systems in Bangladesh.

Cropping systems in Bangladesh are very complex, highly intensive and diverse, and are continuously evolving and changing (Timsina and Connor, 2001). There are three main cropping seasons: (i) aman or kharif or monsoon (also called kharif-2) from June/July to September/October, (ii) rabi or winter from October/November to February/March, and (iii) spring or pre-kharif or pre-monsoon (also called kharif-1) from March/April to June/July (Fig. 1). In kharif-2, rice (called transplanted aman or T. aman) is the predominantly grown crop (> 90% of area), mostly under rainfed conditions. During the dry rabi season a wide range of crops, including rice (called boro), wheat, maize, pulses (chickpea, lentil and field peas), potatoes and oilseeds (e.g., mustard) are grown. In kharif-1, short-duration cultivars of maize, pulses (mungbean, cowpea) and rice (called aus) are grown. Boro and rabi (winter) maize are either fully or partially irrigated, while aman and aus rice and kharif-1 (spring) maize are predominantly rainfed, with some crops applied with partial irrigation. Wheat is also predominantly grown with full irrigation (~ 80%), with remaining 20% under either partial irrigation or strictly rainfed. Thus, rice-rice (R-R), rice-wheat (R-W), and rice-maize (R-M) are the dominant systems, which often include an additional crop such as aus rice or maize in kharif-1 mostly grown under partial irrigation or rainfed conditions. While R-R is the common rotation in tropical and sub-tropical areas with warm climate in Bangladesh and entire South Asia, R-W and R-M rotations are practiced in the sub-tropical areas with mild winters (Timsina and Connor, 2001, Timsina et al., 2010, Timsina et al., 2011).

### 2.2 Calculation of yield potential and yield gaps

Average Ya of rice, maize and wheat for 2010 in Bangladesh were calculated based on Ya data reported over the 2008–2012 time period (BBS, 2013). Yp (for all irrigated crops) and Yw (for rained rice) were simulated using ORYZA (v3) for rice (Bouman et al., 2001), Hybrid-Maize for maize (Yang et al., 2004), and WOFOST for wheat (Wolf et al., 2011). These models were calibrated and validated for Bangladesh based on primary and secondary data for crop phenology, and soil and yield data recorded from well-managed experiments conducted in major agriculture districts (Comilla, Dinajpur, Gazipur, Rajshahi, Rangpur) during 2010 to 2014 (Hossain and Silva, 2013, Islam, 2016). Yw was not simulated for wheat and maize in this study because < 20% of the total area cultivated with these two crops is rainfed. Instead, we estimated Yw of maize and wheat based on existing field and modelling data from literature (Ali et al., 2008, Carberry et al., 2011, Timsina and Humphreys, 2006).

After compilation of all the data, we followed the protocols of the Global Yield Gap Atlas (GYGA) project for estimating the Yg of all crops (www.yieldgap.org, Grassini et al., 2015, van Bussel et al., 2015). Briefly, a number of locations were selected for each crop-water regime combination based on their relative share of the cultivated area for each crop. Selected locations were representative of > 80% of the area cultivated with rice, wheat, and maize in Bangladesh. Yp and Yw were estimated by using long-term measured weather data and dominant soil types and management practices (planting date, variety maturity, plant density). Measured daily weather data (daily maximum and minimum temperature, rainfall and solar radiation) for 1990 to 2010 were retrieved from the Bangladesh Bureau of Meteorology (BBM, 2015). Detailed management and soil data for each selected location were provided by local agronomists and official statistics. Yg was calculated by subtracting Ya from Yp or Yw. Yp, Ya and Yg were upscaled to country level following a bottom-up approach based on crop area distribution and a climate zone scheme (Van Wart et al., 2013, van Bussel et al., 2015).

### 2.3 Cereal supply and demand calculations

**Fig. 2:**
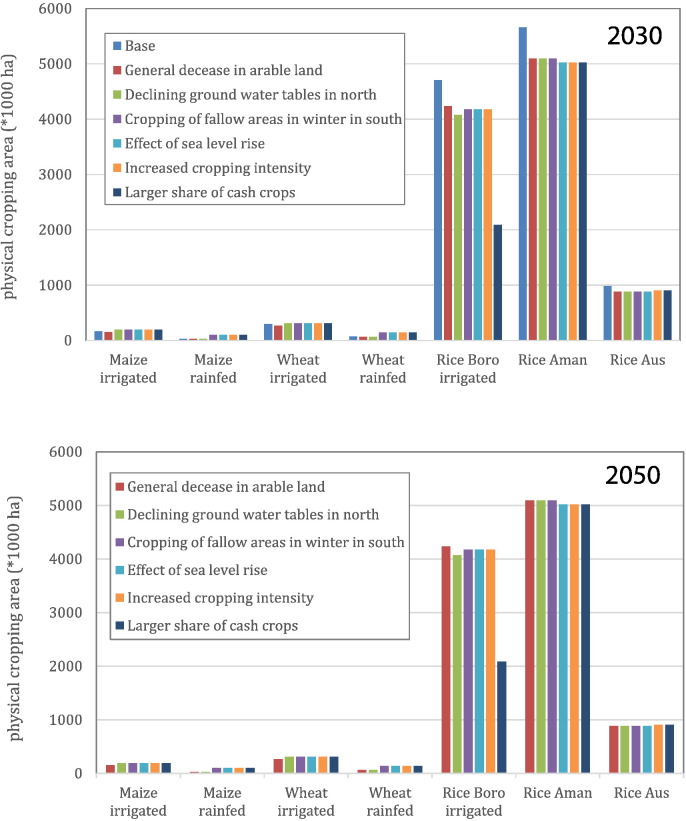
Arable (physical) cropping areas (× 10^3^ ha) in Bangladesh for 2010 and for six cropland change scenarios in 2030 and in 2050.

Domestic supply of each of the three cereals for the base year 2010 was calculated as mean farmers' yield times the harvested area per crop for 2010 (Fig. 2; Table 1) based on data reported by Bangladesh Bureau of Statistics - BBS (BBS, 2013) and FAO (FAOSTAT, 2015). Total maize consumption for Bangladesh in 2010, as estimated by the IMPACT model (Robinson et al., 2015), was very low in comparison to FAO food balance data, and predicted to increase little towards 2050. Since IMPACT maize consumption was estimated based on data collected prior to 2000 (Islam, 2003), it is likely that these estimates have not accounted for the rapid development of the poultry and fish production in Bangladesh during the last decade, resulting in increased demand for maize (Miah et al., 2013). Hence, we decided not to use the IMPACT data for maize but to use the FAO food balance. There was good agreement between IMPACT and FAO food balance data on rice and wheat consumption. Current total cereal demand (expressed in kg milled rice) was calculated by multiplying the 2010 UN population data (UN, 2015) with total demand per capita per cereal (inclusive that for animal and fish feeds) in 2010 (Table 2), derived from the FAO food balance for maize (FAOSTAT, 2015) and from IMPACT for wheat and rice (Robinson et al., 2015). To convert maize and wheat to milled rice equivalents, we used the ratios between the caloric contents of maize and wheat versus that of rice, i.e., 3600 kcal kg^− 1^ milled rice grains, 2730 kcal kg^− 1^ wheat grain and 3180 kcal kg^− 1^ maize grain (FAOSTAT, 2015). Finally, SSR was calculated by dividing the current cereal production by the current cereal food and feed demand, assuming a waste of 15% based on the FAO food balance (FAOSTAT, 2015).

**Table 1:** Current and future population (medium variant)^a^, current and future maize, rice, wheat and all-grains demands per capita (in kg air dry grains)^b^, and total current and future demands^c^, and the relative future changes in annual total grain demand in 2030 and 2050 versus that in 2010 in Bangladesh.

Projections	Population (10^6^)^a^	Annual demand per capita (kg grain air dry)^b^				Change in future annual demand per capita (%)	Annual national demand (M ton grain air dry)^c^				Change in future annual total national all-grains demand (%)
		Maize	Wheat	Rice^d^	All-grains^e^		Maize	Wheat	Rice^d^	All-grains^e^	
2010	151	9.9	25.9	170.5	198.9	–	1.50	3.91	25.76	30.05	–
2030	185	12.5	29.6	162.7	196.2	− 1.36	2.31	5.47	30.11	36.30	+ 20.8
2050	202	14.0	34.7	145.8	184.5	− 7.24	2.83	7.01	29.45	37.26	+ 24.0

a Based on medium fertility population prospect in 2030 and 2050 (UN, 2015).

b Demand per capita in 2010, 2030 and 2050 is based on the current and future demands for wheat and rice as projected by IMPACT and for maize by FAO food balance, and on a combination of current (2010) consumption as estimated by FAO and demand increase in 2030 and 2050 relative to 2010 as estimated by IMPACT for wheat and rice and by FAO for maize (see Section 2.3 for details).

c For the calculation of the total food demand, a loss fraction in food production of 15% on average (source: http://faostat3.fao.org/download/FB/ was assumed.

d Milled rice.

e Milled rice equivalent.

**Table 2:** Scenarios used for land use changes in 2030 and 2050 in Bangladesh.

Scenario	2030	2050
A (general decrease in arable land)	10% decrease in arable area (across the country)	20% decrease in arable area (across the country)
B (declining groundwater tables in north)	Scenario A + 10% decrease in boro rice and 5% increase in rabi maize & wheat areas (in north)	Scenario A + 20% decrease in boro rice and 10% increase in rabi maize & wheat areas (in north)
C (cropping of fallow areas in winter in south)	Scenario B + 40% of fallow areas cropped with boro rice and 30% with maize & wheat (in south)	Scenario B + 40% of fallow areas cropped with boro rice and 30% with maize & wheat (in south)
D (effect of sea level rise)	Scenario C + 5% decrease in aman rice areas due to sea level rise (in south)	Scenario C + 10% decrease in aman rice areas due to sea level rise (in south)
E (increased cropping intensity)	Scenario D + 5% increase in aus rice areas (in south) and 10% increase in kharif-1 maize areas (in north)	Scenario D + 10% increase in aus rice areas (in south) and 20% increase in kharif-1 maize areas (in north)
F (larger share of cash crops)	Scenario E + 50% decrease in boro rice areas ≥ high value crops (across the country)	Scenario E + 50% decrease in boro rice areas ≥ high value crops (across the country)

We chose years 2030 and 2050 for future self-sufficiency assessments because government policy makers need quantitative information to develop strategies to meet future food requirements for both the mid- and long-term. Grain production for each of the three cereals and the total aggregated production (in kg milled rice equivalent) was calculated for 2030 and 2050 by multiplying the future land area for each crop by their estimated yield (Fig. 2). Total annual demand per capita in years 2030 and 2050 for rice and wheat were derived from the IMPACT model while for maize were derived from the FAO food balance, and total grain demands for those years were projected using the future population sizes based on UN population prospects (Table 1). SSRs were calculated for each scenario as the ratio between predicted production and demand, separately for each crop, and also based on the sum of the three crops (‘all-grains’ in milled rice equivalents). In view of the continued increase in population and change in diet, SSRs in 2030 and 2050 were examined for the following two scenarios:

• Change in population size relative to 2010 by using low, medium, and high variant UN population projections.
• Decrease in rice demand and increase in maize demand in 2030 and 2050 relative to the demands in 2010. We assume a decrease in rice consumption by 10% in 2030 and by 20% in 2050 relative to the current rice consumption per capita (i.e. 170.5 kg milled rice person^− 1^ yr^− 1^) and replacement of these decreases in intake by an increase in maize consumption by similar caloric amounts. In an extra variant these decreases in rice consumption were also replaced by a doubled caloric intake in the form of additional maize consumption in both 2030 and 2050 to account for increased demand for feed for livestock and fish.

### 2.4 Scenarios of yield and land use in year 2030 and 2050

We assessed six scenarios, which are described below (Table 2):

• **Scenario A** (general decrease in arable land): In this scenario, arable land areas for all crops in Bangladesh will decrease by 10% in 2030 and by 20% in 2050 relative to that of the base year 2010. These relative changes are based on the actual arable land area decreases between 2002 and 2012 to account for increases in residential housings, industries, roads, and aquaculture (Hasan et al., 2013).
• **Scenario B** (declining groundwater tables in the north): Harvested boro rice areas in 2011–2012 in the northern districts (Dinajpur, Rangpur, Bogra, Pabna, Rajshahi regions) were about 1.61 M ha (BBS, 2013) and are assumed to decrease by 10% in 2030 and by 20% in 2050 due to declining groundwater tables. Half of these ‘decreased’ areas are replaced by rabi maize and wheat (i.e., by 2.5% and 5.0% area increase for each crop in 2030 and 2050, respectively). We assume that the other 5% area in 2030 and 10% in 2050 are used for other crops (e.g. legumes, oilseeds, potatoes, etc.) and aquaculture. Our estimates are based on Dey et al. (2013), Mbugua and Snijders (2012) and Qureshi et al. (2015), who all reported a future reduction in boro rice areas as a result of declining groundwater tables in the northern districts.
• **Scenario C** (cropping of fallow areas in winter in the south): At present, approximately 0.25 M ha land in southern Bangladesh (Barisal, Chittagong, Khulna, Noakhali, Patuakhali regions) remain fallow during winter, which is about 15% of cultivable land in the south (MoA-FAO, 2012). In this scenario we assumed that of this fallow land 0.10 M ha (40%) can potentially be brought under surface irrigation in a short time-frame (MOA-FAO, 2012, Santos Valle et al., 2014) and be used for boro rice during the winter in future. In fact, the government has already planned investment and started implementing infrastructure projects for the coastal region to increase dry season cropping intensity to increase food security (Government of Bangladesh, 2010, MOA-FAO, 2012). Of the remaining 0.15 M ha (60%), 0.075 M ha (30%) can be used for both wheat and rabi maize and can be cultivated under rainfed conditions in future. This scenario is based on a detailed study in southern Bangladesh which concluded that most fallow lands in the south can be used for wheat cultivation by utilizing shallow ground water or irrigation water from ponds during the rabi season (Rawson, 2011). In addition, GoB has identified maize as a high priority crop and aquaculture consuming maize as a potential source of nutrition and income in this region (MoA-FAO, 2012).
• **Scenario D** (effect of sea level rise): In 2012, there was approximately 1.51 M ha of land under aman rice in the southern coastal districts (BBS, 2013). We assume a decrease in aman rice area by 5% in 2030 and by 10% in 2050 due to sea level rise. No effect is assumed on winter crop areas, as sea water will enter the inland areas only in the monsoon season and farmers will strive to grow rabi crops by utilizing shallow groundwater for irrigation, resulting from inundation in monsoon. This scenario was chosen because climate change studies have forecasted significant sea level rise in southern Bangladesh (IPCC, 2014, WARPO, 2006), possibly resulting in inundation and loss of land areas (Clark et al., 2015, MOA-FAO, 2012, WARPO, 2006) and loss of crop production (Clark et al., 2015, Thomas et al., 2012) in future.
• **Scenario E** (increased cropping intensity): In 2012, there was about 0.42 M ha of land with aus rice in the southern districts and 30,000 ha with kharif-1 maize in the northern districts (BBS, 2013). It is assumed there will be an increase in aus rice areas by 5% in 2030 and by 10% in 2050 in the southern districts, which means that cropping intensity will increase from single/double to double/triple to triple/quadruple cropping (Ahmed et al., 2013, BBS (Bangladesh Bureau of Statistics), 2013). Likewise, it is assumed there will be an increase in kharif-1 maize areas by 10% in 2030 and by 20% in 2050 in the northern districts, which also results in an increase in cropping intensity. This scenario was chosen to resemble GoB's current priority to increase aus rice production in southern Bangladesh and maize production all over Bangladesh (Government of Bangladesh, 2010, MOA-FAO, 2012). This scenario is the result of combination of all changes that may occur in future.
• **Scenario F** (larger share of cash crops): In this scenario, we assume that 50% of the boro rice areas in Bangladesh will be used for growing high value crops such as vegetables, spices, pulses and oilseeds, and aquaculture due to shifts in consumption patterns from rice to other crops, vegetables, and meat and fish, and also to meet the demands for export (Alkanda, 2010, Amarasinghe et al., 2014, Mukherjee et al., 2011). This scenario, although hypothetical, was chosen because farmers need to increase their income substantially, for example by cultivating high value crops. Besides, they may have to shift from high water requiring boro rice to less water demanding alternative crops to reduce total water demand, which has also been government's priority (Government of Bangladesh, 2010, MOA-FAO, 2012).

For each of the abovementioned scenarios, we analyzed the consequences of three increasing degrees of Yg closure by year 2030 and 2050:

• No Yg closure: Ya of rice, wheat and maize in 2030 and 2050 were assumed to be identical as in 2010.
• 50% closure of the gap between Ya and 80% of Yw or 85% of Yp, i.e. 50% closure of the so-called exploitable yield gap (see Van Ittersum et al., 2013). In the remainder of this paper we label this ‘50% yield gap closure’.
• Yield gap closure to 85% of Yp (irrigated crops) and 80% of Yw (rainfed crops), labelled ‘full yield gap closure’. These levels represent attainable yields in well-managed farmers' fields (Cassman et al., 2003, Van Ittersum et al., 2013).

## 3 Results

### 3.1 Yield potential and yield gaps

Yield potential of irrigated (boro) rice in Bangladesh was 11.7 t ha^− 1^ while Yw of rainfed aman and aus rice were respectively 6.5 and 7.8 t ha^− 1^ (Table 3). Yp of irrigated maize and wheat were, respectively, 11.4 and 5.5 t ha^− 1^, while Yw of rainfed maize and wheat were respectively 8.0 and 3.0 t ha^− 1^. Yield gaps of rainfed rice, irrigated rice, irrigated maize and irrigated wheat ranged across the country from 44 to 54%, 45–61%, 30–65%, and 47–65% of Yp, respectively (Table 3; Figure SI1). Yield gaps of both rainfed and irrigated rice were higher in northern districts compared to southern districts while those of maize and wheat were higher in southern districts compared to northern districts (Table 3; Figure SI1). Large variations in Yg across the climatic zones indicate large variations in Yp as well as in Ya across those zones (Figure SI2 and Figure SI3), suggesting that there are plenty of opportunities to close the yield gaps of these cereals under both irrigated and rainfed conditions.

**Table 3:** Mean values (t ha^− 1^) for the potential (Yp), water-limited potential (Yw), and actual (Ya - 2010) yields, the yield gaps (Yg), 85% of Yp or 80% of Yw, or full Yg closure, and 50% Yg closure for the years 2030 and 2050 for the main grain crops in Bangladesh.

Yield levels (t ha-1)	Maize		Wheat		Rice		
	Irrigated, rabi	Rainfed, kharif-1 and rabi	Irrigated	Rainfed	Irrigated, boro	Rainfed, aman	Rainfed, aus
Yield potential (irrigated or rainfed; Yp or Yw)	11.40	8.00	5.50	3.00	11.70	6.50	7.80
Actual yield (Ya)	6.20	4.50	2.80	1.80	5.85	3.41	2.88
Yield gap (Yg)	5.20	3.50	2.70	1.20	5.85	3.09	4.92
85% of Yp, or 80% of Yw, or full Yg closure	9.69	6.40	4.68	2.40	9.95	5.20	6.24
50% Yg closure	7.95	5.45	3.74	2.10	7.90	4.31	4.56

### 3.2 Changes in food demand towards 2030 and 2050

With the medium variant of population increase, the combined total demand for wheat, maize and rice (all-grains demand in milled rice equivalent) in 2030 and 2050 are projected to be 21 and 24% higher than in 2010 (Table 1). The projections indicate that although in absolute terms the increases are mostly due to the increase in demand for rice, in relative terms the demand for maize will increase steeply. With the low and high variants of population increase, combined total demand is expected to increase by respective 13 and 29% (year 2030) and 6 and 45% (year 2050) relative to year 2010.

### 3.3 Future production of maize, rice, wheat, and all-grains

With 2010 land areas and yield levels (i.e. base scenario), the total production of maize, wheat, rice, and all-grains in 2010 was 1.0, 0.8, 28.3, and 29.7 million tons, respectively (Table 4). Thus, the maize production could potentially meet two-thirds of the demand, while considerable amount of wheat import was required (Table 1, Table 4). The production of rice and all-grains, was just sufficient to meet the national demand (Table 1, Table 4). When the arable land area decreases by 20% (scenario A) and yield levels remain the same in 2050 as in 2010, the total production of all cereals as well as that of all-grains will also decrease sharply. Scenarios B, C, D and E (but with 2010 yield levels) lead mainly to a small decrease in rice production, but to small increases in maize and wheat production compared to scenario A (Table 4). Halving the boro rice area in scenario F results in strong production decreases for both rice and all-grains (15.8 and 17.8 million tons, respectively) compared to the production under other scenarios (21.6–21.9 million tons for rice and 23.2–23.9 million tons for all-grains). By achieving 80% of Yw or 85% of Yp of all crops, the production of all individual cereals and all-grains will increase significantly for all scenarios, except scenario F in which 50% of boro rice is replaced by cash crops. For scenario F, there will be further reduction in rice and all-grains production (26.0 and 29.1 million tons, respectively), indicating that there will not be enough rice and all-grains even though 80–85% of Yp or Yw is achieved. With 50% yield gap closure, there will be a slight decrease in rice production, strong and moderate increases in respectively, maize and wheat production (Table 4).

**Table 4:** Total production (million tons grains yr^− 1^) for maize, rice, wheat and all-grains for 2010 (with yields and land use for 2010), and for future supply^a^ (with 2010 yield levels, 85% of Yp and 80% of Yw, and 50% yield gap closures) for six land use change scenarios in 2050 in Bangladesh.

Year	Scenarios	Maize	Wheat	Rice	All-grains^b^
2010	Base scenario	1.01	0.83	28.29	29.67

2010 yield levels					
2050	Scenario A	0.81	0.66	22.63	23.74
	Scenario B	1.23	0.86	21.56	23.19
	Scenario C	1.52	0.97	21.89	23.86
	Scenario D	1.52	0.97	21.60	23.57
	Scenario E	1.54	0.97	21.67	23.66
	Scenario F	1.54	0.97	15.77	17.79

80 of Yw or 85% of Yp					
2050	Scenario A	1.25	1.08	37.54	39.28
	Scenario B	1.91	1.40	35.72	38.29
	Scenario C	2.32	1.55	36.28	39.33
	Scenario D	2.32	1.55	35.84	38.88
	Scenario E	2.35	1.55	35.98	39.06
	Scenario F	2.35	1.55	25.95	29.08

50% yield gap closure					
2050	Scenario A	1.03	0.87	30.09	31.51
	Scenario B	1.57	1.13	28.64	30.74
	Scenario C	1.92	1.26	29.09	34.06
	Scenario D	1.92	1.26	28.72	31.40
	Scenario E	1.95	1.26	28.83	31.36
	Scenario F	1.95	1.26	20.86	23.43

a Future cereal supply is calculated as the actual yields (2010), 85% of Yp for irrigated and 80% of Yw for rainfed crops, or 50% Yg closure (2050) times the actual area and yields in 2010 (base scenario), or the future land areas used for the three grain crops for the six land use change scenarios for 2050.

b Total production of the three main grain crops is converted into milled grains (air dry) by applying the ratios between the amount of calories kg^− 1^ grains for the main grain crops and that for milled rice (i.e., maize 3180 kcal kg^− 1^; milled rice 3600 kcal kg^− 1^; wheat 2730 kcal kg^− 1^).

### 3.4 Future cereal self-sufficiency ratios for different scenarios

Current (2010) SSRs for maize and wheat are 0.67 and 0.21 respectively, while for rice it is 1.09, resulting in 0.99 for all-grains (Table 5). For the future scenario without yield increase and no cropland area expansion, SSRs for all the three grain crops as well for the combined in milled rice equivalent decrease considerably in 2030 (Table 5). For all land use change scenarios in 2030 but scenario F, SSR for all-grains decreases from 0.82 to 0.73–0.75 due to decease in SSRs for rice (from 0.94 to 0.82–0.84), while the SSRs for maize increase in most scenarios (from 0.44 to 0.61). Scenario F assumes a drastic decrease of boro rice area, resulting in much lower SSRs for rice (0.60) and all-grains (0.55). As the demand in 2050 is projected to be only moderately higher than in 2030 (Table 1), SSR for all-grains for the base scenario in 2050 will only be slightly lower than that in 2030 (0.80 vs. 0.82; Table 5). As land areas are assumed to further decrease between 2030 and 2050 under scenarios A-F, SSRs for all-grains in 2050 are projected to decrease further, i.e., from 0.80 (baseline) to 0.62–0.64 (scenarios A–E) and 0.48 (scenario F).

**Table 5:** Self-sufficiency ratios for maize, rice, wheat and all-grains in 2010 (i.e., with yield levels and land use of 2010), and for 2030 and 2050^a^ demand^b^ (with 2010 yield levels). Current land use (base scenario) and six different land use change scenarios (scenarios A–F) are assessed.

Year	Scenarios	Maize	Wheat	Rice	All-grains
2010	Base scenario	0.67	0.21	1.09	0.99
2030	Base scenario	0.44	0.15	0.94	0.82
	Scenario A	0.39	0.14	0.84	0.74
	Scenario B	0.49	0.15	0.82	0.73
	Scenario C	0.61	0.18	0.84	0.75
	Scenario D	0.61	0.18	0.83	0.74
	Scenario E	0.61	0.18	0.83	0.74
	Scenario F	0.61	0.18	0.60	0.55
2050	Base scenario	0.36	0.12	0.96	0.80
	Scenario A	0.29	0.10	0.77	0.64
	Scenario B	0.44	0.12	0.73	0.62
	Scenario C	0.54	0.14	0.74	0.64
	Scenario D	0.54	0.14	0.73	0.63
	Scenario E	0.55	0.14	0.73	0.64
	Scenario F	0.55	0.14	0.53	0.48

a Future food supply is calculated as the actual yields times the actual area (in 2010), or the future areas used for the three crops for the six different land use change scenarios for 2030 and 2050. Actual yields are year 2010 yields.

b Future food demand is calculated as the estimated population sizes for 2030 or 2050 from UN medium population projection (source: UN population prospects, see http://esa.un.org/wpp/) times the mean demands per capita in Bangladesh for rice and wheat as derived from the IMPACT and for maize from FAO food balance.

With Yg closure equivalent to 80% of Yw or 85% of Yp on existing cropland area, and cereal demand estimated based on the medium UN variant scenario, estimated SSRs for maize, wheat, rice, and all-grains by 2030 are 0.68, 0.25, 1.55, and 1.35, respectively, indicating that Bangladesh can be self-sufficient for rice and all-grains through Yg closure (Table 6). And even with a 10% decrease in cropland area (scenario A), Bangladesh remains self-sufficient for rice and all-grains by year 2030 (SSRs of 1.40 and 1.22 respectively). Likewise, even with 50% yield gap closure on existing cropland area, SSRs in 2030 for rice and all-grains remains > 1 (1.24 and 1.09, respectively) and with 10% decrease in area, the respective SSRs will be 1.12 and 0.98 (Table 6). However, in all the above mentioned scenarios, SSRs for maize and wheat remain low (< 0.68 and < 0.25 respectively). Still, it is remarkable that with 50% yield gap closure (i.e., an increase in Ya of around 25% for maize and wheat and around 35–40% for rice between 2010 and 2030), Bangladesh can maintain an overall cereal SSR close to one.

**Table 6:** Self-sufficiency ratios for maize, rice, wheat and all-grains for future supply^a^ (85% of Yp for irrigated and 80% of Yw for rainfed crops, and 50% yield gap closure) and demand^b^ situations for the current land use and for six different land use change scenarios for 2030 in Bangladesh.

Scenarios	Maize	Wheat	Rice	All-grains
80 of Yw or 85% of Yp				
2010 area	0.68	0.25	1.55	1.35
Scenario A	0.61	0.22	1.40	1.22
Scenario B	0.75	0.25	1.37	1.20
Scenario C	0.93	0.28	1.38	1.23
Scenario D	0.93	0.28	1.38	1.23
Scenario E	0.94	0.28	1.38	1.23
Scenario F	0.94	0.28	0.99	0.91

50% yield gap closure				
2010 area	0.58	0.20	1.24	1.09
Scenario A	0.50	0.18	1.12	0.98
Scenario B	0.62	0.20	1.10	0.97
Scenario C	0.77	0.23	1.11	0.99
Scenario D	0.77	0.23	1.10	0.98
Scenario E	0.78	0.23	1.11	0.99
Scenario F	0.78	0.23	0.80	0.73

a Future food supply is calculated as full yield gap closure (85% of Yp for irrigated and 80% of Yw for rainfed crops) and 50% yield gap closure (for 2030) times the actual area (in 2010) and the future areas used for the three grain crops for the six different land use change scenarios for 2030.

b Future food demand is calculated as the estimated population sizes for 2030 from UN medium population projection (source: UN population prospects, see http://esa.un.org/wpp/) times the mean demands per capita in Bangladesh for rice and wheat as derived from the IMPACT and for maize from FAO food balance.

For scenarios B–E, there are higher SSRs for maize, wheat and all-grains compared to scenario A with both 50% yield gap closure (0.18–0.98 for scenario A vs. 0.20–0.99 for others) and full closure (0.22–1.22 vs. 0.28–1.23). A 50% decrease in boro rice areas (scenario F) leads to SSRs < 1 for all crops (0.23–0.80 for 50% Yg closure and 0.28–0.99 for full yield gap closure) (Table 6).

Because land areas are assumed to continue to decrease between 2030 and 2050, SSRs for all-grains under all scenarios except scenario F in 2050 may drop to values just above 1.0 (from 1.32 to 1.03–1.06) with 80% of Yw or 85% of Yp), while the levels drop to well below 1.0 (from 1.06 to 0.83–0.88) with only 50% yield gap closure. For scenario F, SSRs drop to 0.78 and 0.63, respectively, with full or 50% yield gap closures. For rice, SSRs for various scenarios, except scenario F, will range from 1.21 to 1.27 with full yield gap closures and from 0.97 to 1.02 with 50% yield gap closure, while for maize and wheat they range from 0.36 to 0.83 and from 0.12 to 0.22 across the two levels of Yg closures. For scenario F, SSRs for the three cereals range from 0.18 to 0.70 with 50% yield gap closure and from 0.22 to 0.88 with full yield gap closure (Table 7). The SSR values with < 1.0 for scenario F in both 2030 and 2050 indicate that, even a yield gap closure with 80% of Yw or 85% of Yp will not be sufficient to meet cereal demand, including rice, if a drastic decrease in boro rice area occurs in future.

**Table 7:** Self-sufficiency ratios for maize, rice, wheat and all-grains for future supply^a^ (85% of Yp for irrigated and 80% of Yw for rainfed crops, and 50% yield gap closures) and demand^b^ situations for the current land use (i.e. 2010 area) and for six different land use change scenarios for 2050 in Bangladesh.

Scenarios	Maize	Wheat	Rice	All-grains
80 of Yw or 85% of Yp				
2010 area	0.55	0.19	1.59	1.32
Scenario A	0.44	0.15	1.27	1.05
Scenario B	0.68	0.20	1.21	1.03
Scenario C	0.82	0.22	1.23	1.06
Scenario D	0.82	0.22	1.21	1.04
Scenario E	0.83	0.22	1.22	1.05
Scenario F	0.83	0.22	0.88	0.78

50% yield gap closure				
2010 area	0.46	0.16	1.27	1.06
Scenario A	0.36	0.12	1.02	0.85
Scenario B	0.56	0.16	0.97	0.83
Scenario C	0.68	0.18	1.00	0.85
Scenario D	0.68	0.18	0.97	0.84
Scenario E	0.69	0.18	0.97	0.88
Scenario F	0.71	0.18	0.70	0.63

a Future food supply is calculated as full yield gap closure (85% of Yp for irrigated and 80% of Yw for rainfed crops) and 50% of yield gap closure (for 2050) times the actual area (in 2010) and the future areas used for the three grain crops for the six different land use change scenarios for 2050.

b Future food demand is calculated as the estimated population sizes for 2050 from UN medium population projection (source: UN population prospects, see http://esa.un.org/wpp/) times the mean demands per capita in Bangladesh for rice and wheat as derived from the IMPACT and for maize from FAO food balance.

### 3.5 Effects of different population projections and dietary changes

**Fig. 3:**
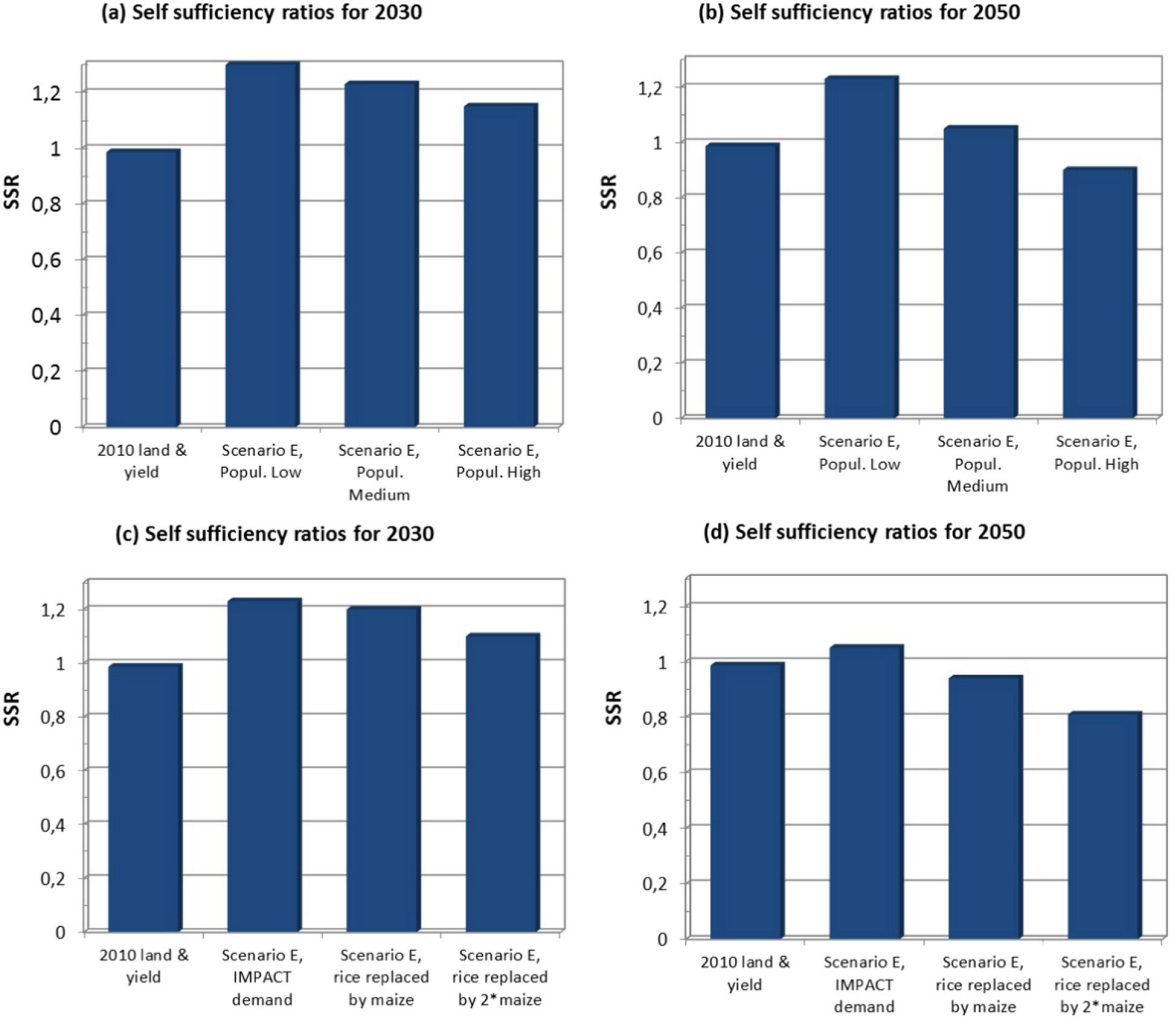
Self-sufficiency ratios (SSRs) for the total supply and the total demand for rice, maize and wheat grains for the base year 2010 (i.e. yields, land use and food demand for 2010) and for land use change scenario E with full yield gap closure (i.e., 80 of Yw or 85% of Yp) and food demands in 2030 (a) and 2050 (b) for respectively low, medium and high population projections, and the same demands but with rice demand per capita decreased by 10% in 2030 (c) and by 20% in 2050 (d) in comparison to the rice demand for 2010 and replaced by the same and the doubled caloric amount as maize grains.

The effect of different variants in population growth for what we consider the most likely scenario, i.e. scenario E with increased cropping intensity, is small for 2030 (Fig. 3a), but substantial for 2050 (Fig. 3b). By 2050, even with Yg closure up to 85% of Yp or 80% of Yw, SSR for this scenario may drop below one under the high population growth variant, because of a combination of high population growth and land use change (Fig. 3b). Changes in relative demands for rice (decrease) and maize (increase) for the medium variant population have a clear effect on cereal self-sufficiency when cereals supplies are calculated under full Yg closure (Fig. 3c, d). This is particularly true for the year 2050 (Fig. 3d), when maize consumption is assumed to double due to increased demand for fish, livestock and poultry products. The SSR for all-grains becomes low (0.94) and further decreases (0.81) for the cases with rice consumption substituted by, respectively, the same or double the amount of maize calories (Fig. 3c, d).

## 4 Discussion and conclusions

Future domestic supply of cereals depends mainly on possible changes in land use and yield gap closure. Our simulation results reveal that the Yp of maize and boro rice in Bangladesh can be > 11 t ha^− 1^ and that of wheat can be > 5 t ha^− 1^, while Yw can range between 6.5 and 7.8 t ha^− 1^ for aman and aus rice (Table 3; Figure SI1 and Figure SI2). The simulated maize Yp in this study is lower than those reported by Timsina et al., 2010, Timsina et al., 2011, the simulated Yp of rice is similar. Likewise, the simulated Yp for wheat across the country is slightly lower than that recorded from well-managed trials from large numbers of fields (Rawson et al., 2011) as well as also lower than those reported by Timsina and Humphreys (2006). The discrepancies in simulated and observed yields for rice are expected as the Yp for boro rice in this study was averaged for eight locations of Bangladesh using observed weather data while the Yp from Timsina et al., 2010, Timsina et al., 2011 was based on only three locations and simulated using the NASA weather data. Difference in Yp for wheat between the current study and Timsina and Humphreys (2006) is probably due to difference in weather years and models used for simulations. We used the WOFOST model while Timsina and Humphreys (2006) used CERES-Wheat and these two studies used different years of weather data for simulations.

The absolute yield gaps of all cereals in this study were large (Table 1). In relative terms, Yg was about 45% for maize and wheat, around 50% for *boro* and *aman*, and > 60% for *aus* rice. Khan et al. (2013) and Mondal (2011) reported 16–60% Yg in rice; Hasan and Islam (2010), Kashem et al. (2012), and Mondal (2011) reported 16–27% for wheat; and Ali et al. (2008) estimated 30–40% Yg in maize. All these authors, except Schulthess et al. (2012), however, calculated Yg as the difference between yields from the highest-yielding treatments in experiments and Ya averaged over 1–2 years while in our study Yg was calculated as the difference between model-based Yp or Yw and Ya, the latter averaged over at least five years across climatic zones; Figure SI3. Actual yields vary by year, season and locations, and thus such discrepancies in Yg across crops and regions are (Grassini et al., 2015). Our Yp, Yw and Yg estimates indicate that the Yg in all cereals can be closed substantially either by increasing the Yp or Ya. Fischer et al., 2009, Fischer et al., 2014 suggested two avenues to close the Yg by: (i) increasing actual farmers' yields more in line with current Yp levels by improving the agronomic management practices such as improved crop, water, nutrient and pest and disease management practices, and (ii) maintaining or increasing the rates of progress of Yp by either adopting modern plant breeding and molecular techniques such as use of molecular markers and new transgenes.

Our estimates indicate that current yields in Bangladesh are not sufficient to meet future food demand due to a combination of expected cropland area reduction and expected grain demand increases. As a result, under various land use change scenarios, SSRs of all cereals will decrease substantially in 2030 and 2050. However, with full yield gap closure (i.e., 80 of Yw or 85% of Yp) rice and all-grains supply will be sufficient (26.0–37.5 and 29.1–39.3 million tons, respectively) to meet their demands (29.5 and 37.3 million tons, respectively) in 2050. However, still some shortage of maize and huge shortage of wheat occurs in the various scenarios (i.e., 1.3–2.4 and 1.1–1.6 million tons, respectively) compared to the demands of 2.8 (maize) and 7.0 (wheat) million tons. With the 50% Yg closure there will be some shortage of rice, but huge shortages of maize, wheat and all-grains.

Our analysis indicates that with the current (2010) yield levels but with various land use change scenarios in 2050, only about 50% of maize demand will be met. If yield gaps are closed to 50% of Yp about 65% of the maize demand will be met, if yield gaps are closed to 85% of Yp, about 85% of maize demand will be met. For wheat, even with full yield gap closure, only about 25% of the demand will be met. Hence, for various land use change scenarios, some imports are required for maize (0.9–1.8 million tons with 50% Yg closure and 0.5–1.6 million tons with full Yg closure) but large amounts for wheat (5.8–6.1 million tons with 50% Yg closure and 5.5–5.9 million tons with full Yg closure). For rice and current yield levels, there will be huge deficit of about 8 million tons yr^− 1^ in 2050.With 50% Yg closure, rice demand will not be fully fulfilled in most scenarios, but with full Yg closure, all rice demand will be met with a surplus of about 6–8 million tons across the scenarios, except for scenario F (larger share of cash crops). The latter scenario requires an import of about 8.5 million tons with 50% closure and about 3.5 million tons with full closure. The reduced boro rice areas under scenario F can be used for maize, wheat, or other high value crops such as spices and vegetables considering comparative advantages of growing crops in different agro-ecological regions so that farmers' income can be increased. We conclude that full Yg closure (i.e., attaining 80 of Yw or 85% of Yp) of all cereals is needed to compensate for area decreases and demand increases, and to maintain self-sufficiency in rice and all-grains jointly.

Our study considers various scenarios related to land use changes and different levels of yield increases or yield gap closure in future. There are also possibilities of happening some or all the characteristics of various scenarios in various combinations. However, to simplify the calculations and draw clear conclusions to help policy makers, we considered only the most likely scenarios in future. Further, although the study indirectly considers the diet changes by allowing increase in area under maize and other high-value crops through reduction in boro rice areas under scenario F, it does not explicitly consider different scenarios of diet changes such as from cereals to meat- and fish-based products. In addition, it is likely that crop production in Bangladesh will be negatively affected by climate change due to its location in a delta, being prone to storms surges, sea level rise and flooding hazards (Thomas et al., 2012). Hence, we believe that our projections might be optimistic in the light of climate change. Yet, our analysis provides a robust basis for the assessment of food self-sufficiency and food security for Bangladesh under additional scenarios of climate.

## Appendix A Supplementary data

Supplementary figures Image 1
