# Supplementary material for: Can Bangladesh produce enough cereals to meet future demand?
Source: Agric Syst. 2018 Jun;163:36–44. doi: 10.1016/j.agsy.2016.11.003 (PMC5903259; doi:10.1016/j.agsy.2016.11.003)
Supplement: Supplementary file 1 — Supplementary figures [file mmc1.docx]

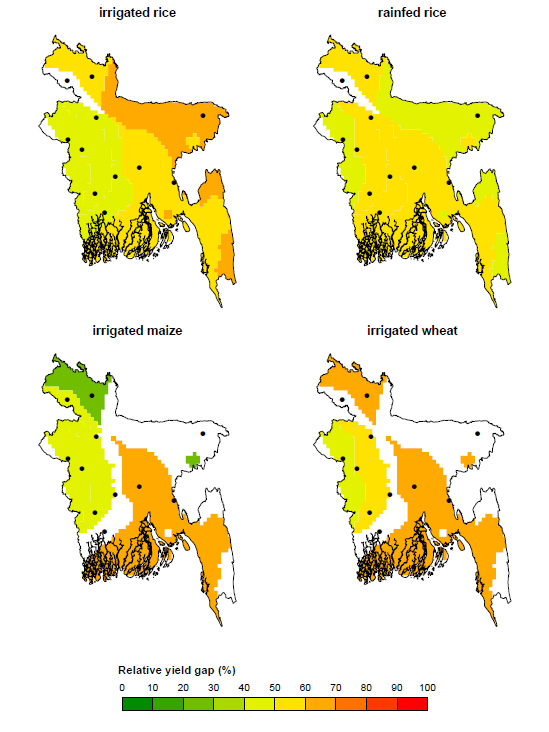


Figure SI1 : Relative yield gap (%) of rice, wheat and maize across climatic zones in Bangladesh.


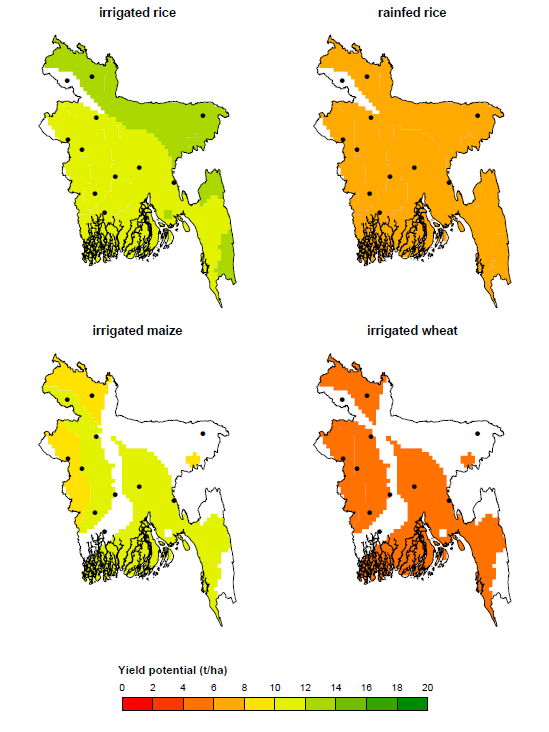


Figure SI2 : Yield potential (t/ha) of rice, wheat and maize across climatic zones in Bangladesh.


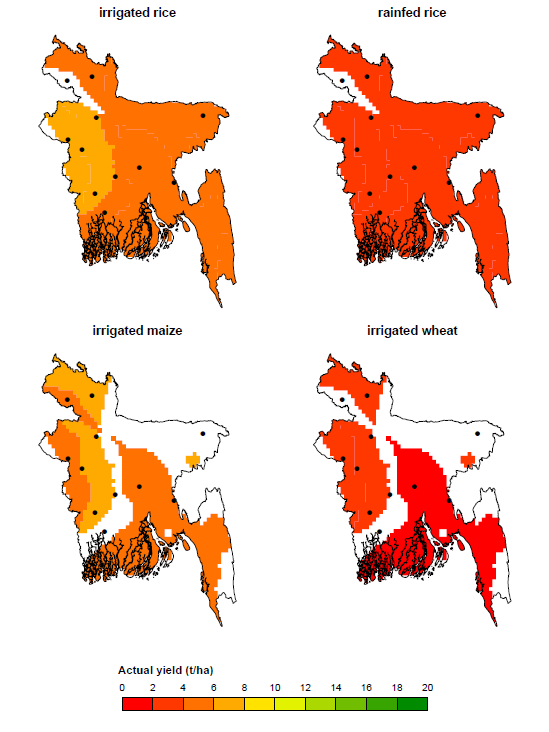


Figure SI3 : Actual yield (t) of rice, wheat and maize across climatic zones in Bangladesh.
